# Crystal structure, Hirshfeld surface analysis and corrosion inhibition study of 3,6-bis­(pyridin-2-yl)-4-{[(3a*S*,5*S*,5a*R*,8a*R*,8b*S*)-2,2,7,7-tetra­methyl­tetra­hydro-5*H*-bis­[1,3]dioxolo[4,5-*b*:4′,5′-*d*]pyran-5-yl)meth­oxy]meth­yl}pyridazine monohydrate

**DOI:** 10.1107/S2056989019009848

**Published:** 2019-07-12

**Authors:** Mouad Filali, Hicham Elmsellem, Tuncer Hökelek, Abdelkrim El-Ghayoury, Oleh Stetsiuk, El Mestafa El Hadrami, Abdessalam Ben-Tama

**Affiliations:** a Laboratoire de Chimie Organique Appliquée, Université Sidi Mohamed Ben Abdallah, Faculté des Sciences et Techniques, Route d’Immouzzer, BP 2202, Fez, Morocco; b Laboratoire de Chimie Analytique Appliquée, Matériaux et Environnement (LC2AME), Faculté des Sciences, BP 717, 60000 Oujda, Morocco; c Department of Physics, Hacettepe University, 06800 Beytepe, Ankara, Turkey; d Laboratoire MOLTECH-Anjou, UMR 6200, CNRS, UNIV Angers 2 bd Lavoisier, 49045 Angers Cedex, France

**Keywords:** crystal structure, pyridazine, dioxolo, Hirshfeld surface, electrochemical measurements

## Abstract

In the title compound, C_27_H_30_N_4_O_6_·H_2_O, the two dioxolo rings are in envelope conformations, while the pyran ring is in a twisted-boat conformation. The pyradizine ring is oriented at dihedral angles of 9.23 (6) and 12.98 (9)° with respect to the pyridine rings, while the dihedral angle between the two pyridine rings is 13.45 (10)°. In the crystal, O—H_water_⋯O_pyran_, O—H_water_⋯O_meth­oxy­meth­yl_ and O—H_water_⋯N_pyridazine_ hydrogen bonds link the mol­ecules into chains along [010]. In addition, weak C—H_dioxolo_⋯O_dioxolo_ hydrogen bonds and a weak *C*—H_meth­oxy­meth­yl_⋯π inter­action complete the three-dimensional structure. The Hirshfeld surface analysis of the crystal structure indicates that the most important contributions for the crystal packing are from H⋯H (55.7%), H⋯C/C⋯H (14.6%), H⋯O/O⋯H (14.5%) and H⋯N/N⋯H (9.6%) inter­actions. Hydrogen-bonding and van der Waals inter­actions are the dominant inter­actions in the crystal packing. Electrochemical measurements are also reported.

## Abstract 2

The title compound is built up by two dioxolo, two pyridine, one pyridazine and one pyran rings. The two dioxolo rings are in envelope conformations, while the pyran ring is in twisted-boat conformation. The pyradizine ring is oriented at dihedral angles of 9.23 (6) and 12.98 (9)° with respect to the pyridine rings, while the dihedral angle between the two pyridine rings is 13.45 (10)°. In the crystal, C—H_dioxolo_⋯O_dioxolo_, O—H_water_⋯O_pyran_, O—H_water_⋯O_meth­oxy­meth­yl_ and O—H_water_⋯N_pyridazine_ hydrogen bonds link the mol­ecules into a supra­molecular structure. A weak C—H_meth­oxy­meth­yl_⋯π inter­action is also observed.

## Chemical context   

Given their importance in the pharmaceutical, chemical and industrial fields, the synthesis of 3,6-di(pyridin-2-yl)pyridazine and its derivatives has been a goal of chemists in recent years. 5-[3,6-Di(pyridin-2-yl)pyridazine-4-yl]-2′-de­oxy­uridine-5′-*O*-triphosphate can be used as a potential substrate for fluorescence detection and imaging of DNA (Kore *et al.*, 2015 ▸). Systems containing this moiety have also shown remarkable corrosion inhibitory (Khadiri *et al.*, 2016 ▸). Heterocyclic mol­ecules such as 3,6-bis (2′-pyrid­yl)-1,2,4,5-tetra­zine have been used in transition-metal chemistry (Kaim & Kohlmann, 1987 ▸). This bidentate chelate ligand is popular in coordination chemistry and complexes of a wide range of metals, including iridium and palladium (Tsukada *et al.*, 2001 ▸). We report herein the synthesis and the mol­ecular and crystal structures of the title compound, (I), along with the Hirshfeld surface analysis and its corrosion inhibition properties.

## Structural commentary   

**Figure 1:**
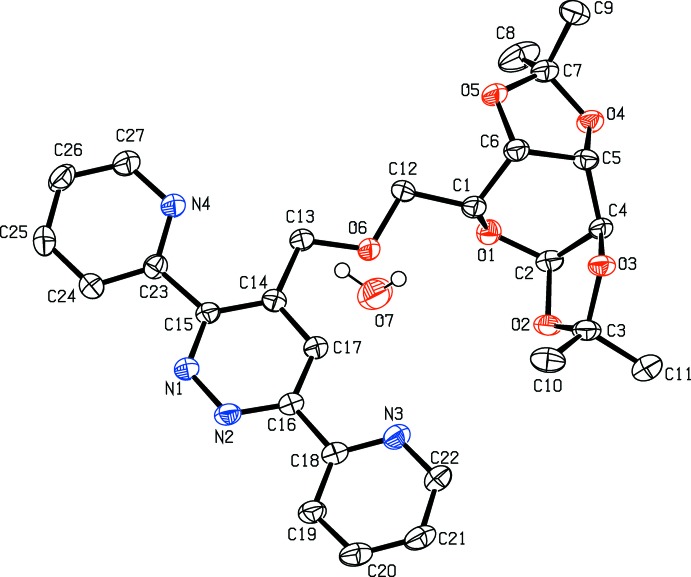
The mol­ecular structure of the title compound with the atom-numbering scheme. Displacement ellipsoids are drawn at the 50% probability level. H atoms bonded to C atoms are not shown.

The title mol­ecule contains two dioxolo, two pyridine, one pyridazine and one pyran rings (Fig. 1 ▸). The pyridazine ring is linked to the pyran ring through the meth­oxy­methyl moiety. The two dioxolo rings, *B* (O2/O3/C2–C4) and *C* (O4/O5/C5–C7), are in envelope conformations. Atoms O3 and O4 are at the flap positions and are displaced by 0.442 (2) and −0.397 (2) Å, respectively, from the least-squares planes of the four atoms. A puckering analysis of the pyran ring *A* (O1/C1/C2/C4–C6), gave the parameters *Q*_T_ = 0.6508 (25) Å, *q*_2_ = 0.6451 (25) Å, *q*_3_ = −0.0865 (26) Å, φ = 214.6 (2)° and θ = 97.64 (23)°, indicating a twisted-boat conformation. The pyradizine ring *D* (N1/N2/C14–C17) is oriented at dihedral angles of 9.23 (6) and 12.98 (9)°, respectively, to the pyridine rings *E* (N3/C18–C22) and *F* (N4/C23–C27), while the dihedral angle between the two pyridine rings is 13.45 (10)°. The meth­oxy­methyl moiety is nearly co-planar with the pyradizine ring, as indicated by the O6—C13—C14—C15 torsion angle of −172.8 (2)°.
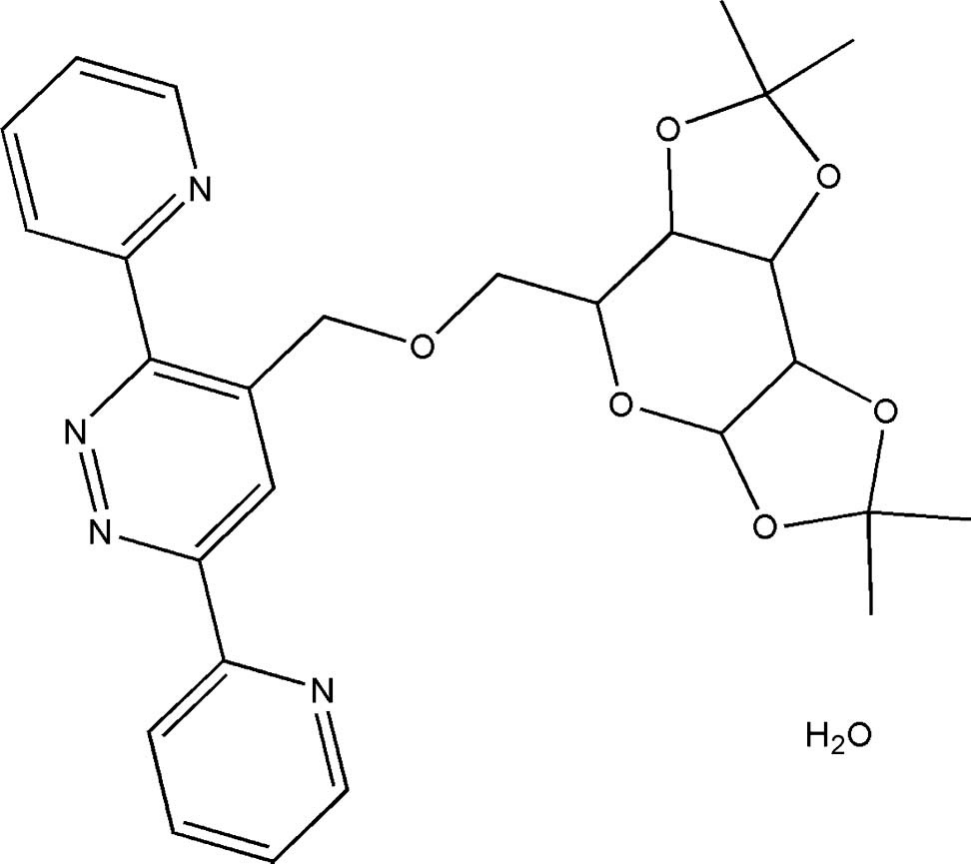


## Supra­molecular features   

**Figure 2:**
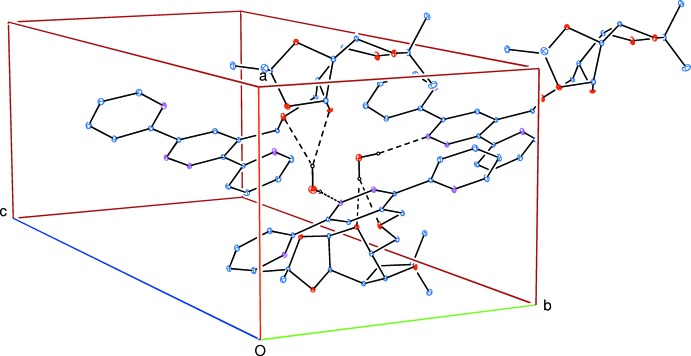
A partial packing diagram showing the O—H_water_⋯O_pyran_, O—H_water_⋯O_meth­oxy­meth­yl_ and O—H_water_⋯N_pyridazine_ hydrogen bonds (Table 1 ▸) as dashed lines.

In the crystal, O—H_water_⋯O_pyran_, O—H_water_⋯O_meth­oxy­meth­yl_ and O—H_water_⋯N_pyridazine_ hydrogen bonds (Table 1 ▸ and Fig. 2 ▸) link the mol­ecules, forming chains along [010]. The hydrogen bond involving H7*B* is bifurcated. In addition, weak C—H_dioxolo_⋯O_dioxolo_ hydrogen bonds and a weak C—H_meth­oxy­meth­yl_⋯π inter­action complete the three-dimensional structure.

## Hirshfeld surface analysis   

**Figure 3:**
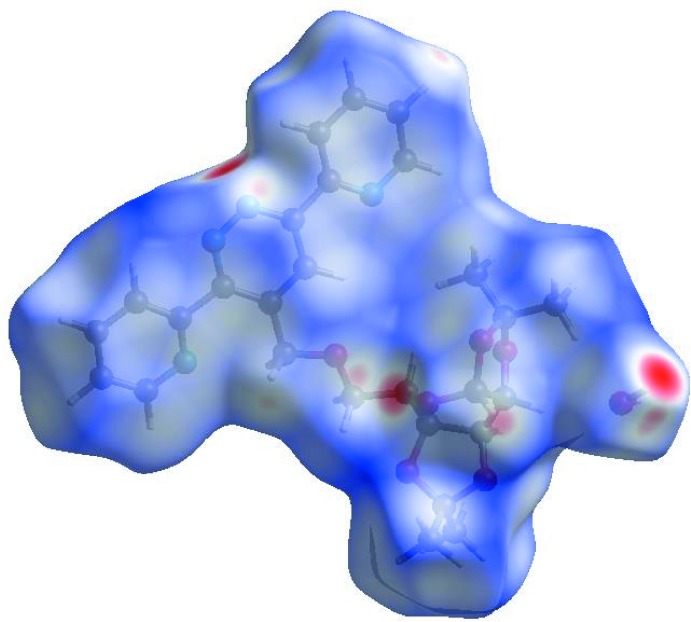
View of the three-dimensional Hirshfeld surface of the title compound plotted over *d*_norm_ in the range −0.4555 to 1.4860 a.u.

**Figure 4:**
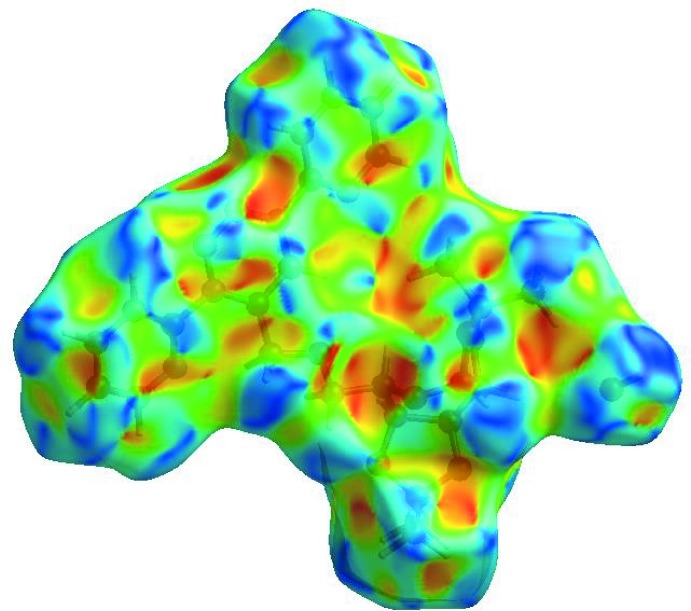
Hirshfeld surface of the title compound plotted over shape-index.

**Figure 5:**
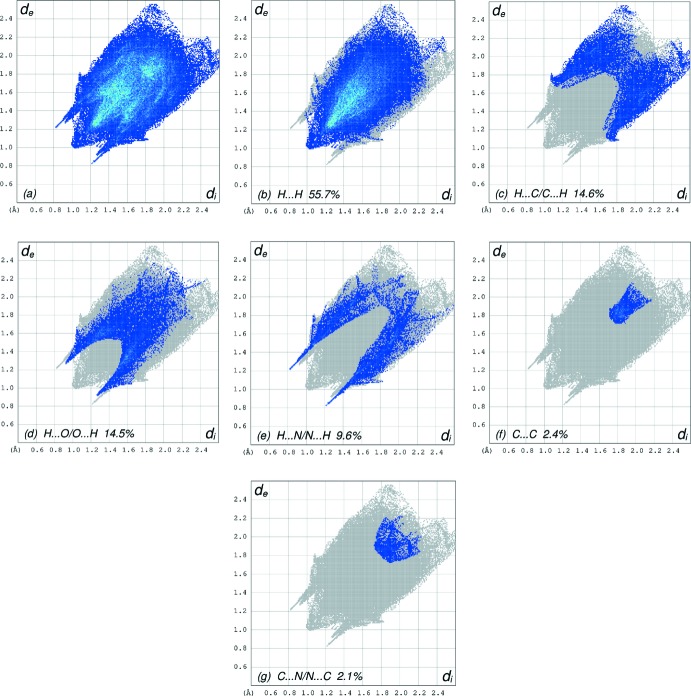
The full two-dimensional fingerprint plots for the title compound, showing (*a*) all inter­actions, and delineated into (*b*) H⋯H, (*c*) H⋯C/C⋯H, (*d*) H⋯O/O⋯H, (*e*) H⋯N/N⋯H, (*f*) C⋯C and (*g*) C⋯N/N⋯C inter­actions. The *d*_i_ and *d*_e_ values are the closest inter­nal and external distances (in Å) from given points on the Hirshfeld surface.

In order to visualize the inter­molecular inter­actions in the crystal of the title compound, a Hirshfeld surface (HS) analysis (Hirshfeld, 1977 ▸; Spackman & Jayatilaka, 2009 ▸) was carried out by using *CrystalExplorer17.5* (Turner *et al.*, 2017 ▸). In the HS plotted over *d*_norm_ (Fig. 3 ▸), white indicates contacts with distances equal to the sum of van der Waals radii, while red and blue indicate distances shorter (in close contact) or longer (distinct contact) than the van der Waals radii, respectively (Venkatesan *et al.*, 2016 ▸). The bright-red spots appearing near O1, O6, N2 and hydrogen atoms H2, H7*A*, H7*B* indicate their roles as the respective donors and/or acceptors. The shape-index of the HS is a tool to visualize the π–π stacking by the presence of adjacent red and blue triangles; if these are absent, then there are no π–π inter­actions. Fig. 4 ▸ clearly suggest that there are no π–π inter­actions in (I). The overall two-dimensional fingerprint plot, Fig. 5 ▸ *a*, and those delineated into H⋯H, H⋯C/C⋯H, H⋯O/O⋯H, H⋯N/N ⋯H, C⋯C and C⋯N/N⋯C contacts (McKinnon *et al.*, 2007 ▸) are illustrated in Fig. 5 ▸ *b*–*g*, respectively, together with their relative contributions to the Hirshfeld surface. Selected contacts are listed in Table 2 ▸.

The most important inter­action is H⋯H, contributing 55.7% to the overall crystal packing, which is reflected in Fig. 5 ▸ *b* as widely scattered points of high density due to the large hydrogen content of the mol­ecule with the tip at *d*_e_ = *d*_i_ ∼1.00 Å. In the presence of a weak C—H⋯π inter­action, the wings in the fingerprint plot delineated into H⋯C/C⋯H contacts (14.6% contribution to the HS) have a symmetrical distribution of points, Fig. 5 ▸ *c*, with the thin and thick edges at *d*_e_ + *d*_i_ = 2.85 and 2.78 Å. The pair of characteristic wings in the fingerprint plot delineated into H⋯O/O⋯H contacts (14.5%, Fig. 5 ▸ *d*) arises from the O—H⋯O and C—H⋯O hydrogen bonds (Table 1 ▸) as well as from the H⋯O/O⋯H contacts (Table 2 ▸) and has a pair of spikes with the tips at *d*_e_ + *d*_i_ = 2.18 Å. The pair of characteristic wings in the fingerprint plot delineated into H⋯N/N⋯H contacts (Fig. 5 ▸ *e*, 9.6%) arises from the O—H⋯N hydrogen bonds (Table 1 ▸) as well as from the H⋯N/N⋯H contacts has a pair of spikes with the tips at *d*_e_ + *d*_i_ = 2.04 Å. Finally, the C⋯C contacts (Fig. 5 ▸ *g*, 2.4%) have a wide spike with the tip at *d*_e_ = *d*_i_ = 1.75 Å.

**Figure 6:**
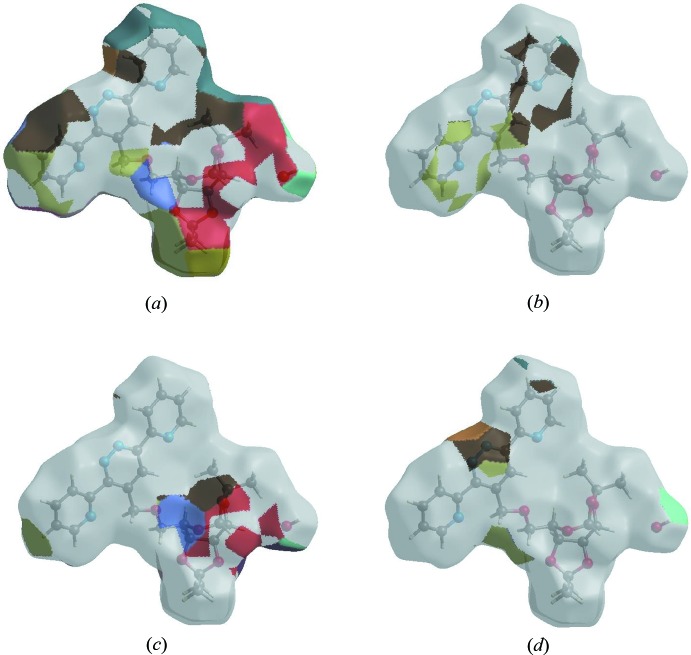
Hirshfeld surface representations with the function *d*_norm_ plotted onto the surface for (*a*) H⋯H, (*b*) H⋯C/C⋯H, (*c*) H⋯O/O⋯H and (*d*) H⋯N/N⋯H inter­actions.

The Hirshfeld surface representations with the function *d*_norm_ plotted onto the surface are shown for the H⋯H, H⋯C/C⋯H, H⋯O/O⋯H and H⋯N/N⋯H inter­actions in Fig. 6 ▸ *a*–*d*, respectively.

The Hirshfeld surface analysis confirms the importance of H-atom contacts in establishing the packing. The large number of H⋯H, H⋯C/C⋯H, H⋯O/O⋯H and H⋯N/N⋯H inter­actions suggest that van der Waals inter­actions and hydrogen bonding play the major roles in the crystal packing (Hathwar *et al.*, 2015 ▸).

## Electrochemical measurements   

**Figure 7:**
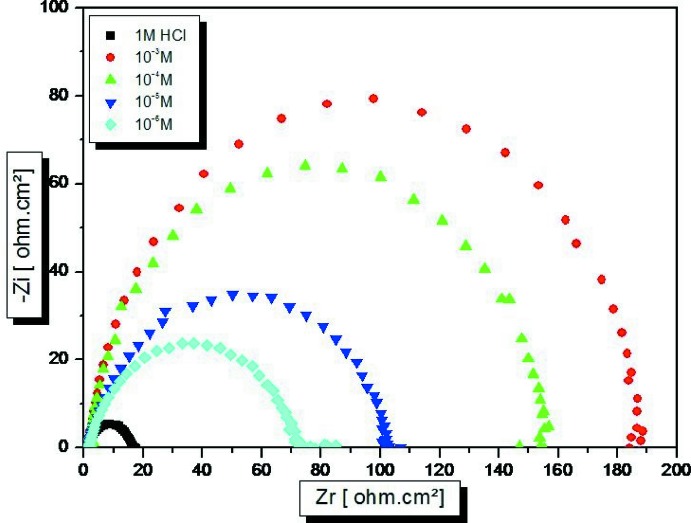
Nyquist plots of mild steel in 1*M* HCl in presence of different concentrations of 3,6-bis­(pyridin-2-yl)-4-{[(3a*S*,5*S*,5a*R*,8a*R*,8b*S*)-2,2,7,7-tetra­methyl­tetra­hydro-5*H*-bis­[1,3]dioxolo[4,5-*b*:4′,5′-*d*]pyran-5-yl)meth­oxy]meth­yl}pyridazine monohydrate.

The effect of the title compound as an inhibitor of the corrosion of mild steel (MS) were studied using electrochemical impedance spectroscopy in the concentration range of 10^−6^ to 10^−3^ *M* at 308 K. The electrochemical experiment consisted of a 3 electrode electrolytic cell consisting of platinum foil as counter-electrode, saturated calomel as reference electrode and MS as working electrode with an exposed area of 1 cm^2^. The MS specimen was immersed in a test solution for 0.5 h until a steady-state potential was achieved using a PGZ100 potentiostat (Bouayad *et al.*, 2018 ▸). Electrochemical impedance spectroscopy (EIS) measurements were performed over a frequency range of 0.1 × 10^−3^ KHz to 10 mHz and an amplitude of 10 mV with 10 points per decade. The percentage inhibition efficiency is calculated from *R*_t_ values as (Sikine *et al.*, 2016 ▸) *E* (%) = [1 − *R*_t(HCl)_/R_t(inh)_] × 100, where *R*_t(inh)_ and *R*_t(HCl)_ are the charge-transfer resistances for MS immersed in HCl, with the title compound and without inhibitor. Nyquist representations of mild steel in 1 *M* HCl in the absence and presence of the inhibitor system are shown in Fig. 7 ▸.

**Figure 8:**
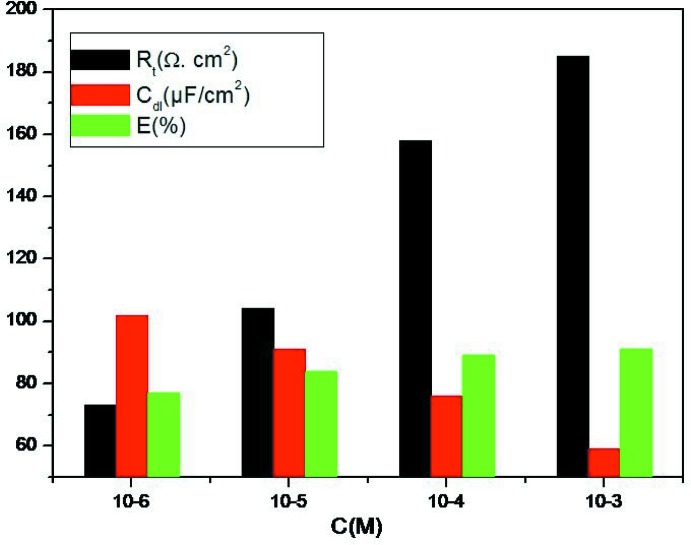
EIS parameters for the corrosion of mild steel in 1*M* HCl with and without inhibitor 3,6-bis­(pyridin-2-yl)-4-{[(3a*S*,5*S*,5a*R*,8a*R*,8b*S*)-2,2,7,7-tetra­methyl­tetra­hydro-5*H*-bis­[1,3]dioxolo[4,5-*b*:4′,5′-*d*]pyran-5-yl)meth­oxy]meth­yl}pyridazine monohydrate at 308 K.

The impedance method provides information about the kinetics of the electrode processes and the surface properties of the investigated systems. The technique is based on the measurement of the impedance of the double layer at the MS/solution inter­face, and represents the Nyquist plots of mild steel (MS) specimens in 1 *M* HCl without and with various concentrations of the inhibitor. The impedance diagrams obtained have an almost semicircular appearance. This indicates that the corrosion of mild steel in aqueous solution is mainly controlled by a charge-transfer process. The imp­edance parameters are given in Fig. 8 ▸. It is observed from the plots that the impedance response of mild steel was significantly changed after addition of the inhibitor. *R*_ct_ is increased to a maximum value of 185 Ω cm^2^ for the inhibitor, showing a maximum inhibition efficiency of 91% at 10^−3^ *M*. The decrease in *C*_dl_ from the HCl acid value of 200 µF cm^−2^, may be due to the increase in the thickness of the electrical double layer or to a decrease in the local dielectric constant (Elmsellem *et al.*, 2014 ▸). This is caused by the gradual displacement of water mol­ecules by the adsorption of organic mol­ecules on the mild steel surface (Hjouji *et al.*, 2016 ▸). Apart from the experimental impedance (EIS) results, the following conclusion is drawn: the alternating impedance spectrum reveals that the double-layer capacitances decrease with respect to the blank solution when the title compound is added. This fact confirms the adsorption of inhibitor mol­ecules on the surface of the MS.

## Database survey   

Silver(I) complexes coordinated by 3,6-di(pyridin-2-yl)pyridazine ligands have been reported (Constable *et al.*, 2008 ▸). Three other metal complexes including 3,6-di(pyridin-2-yl)pyridazine have also been reported, *viz*. aqua­bis­[3,6-bis(pyridin-2-yl)pyridazine-κ_2_ *N* ^1^,*N* ^6^]copper(II) bis­(tri­fluoro­meth­ane­sulfonate) (Showrilu *et al.*, 2017 ▸), tetra­kis­[μ-3,6-di(pyridin-2-yl)pyridazine]bis­(μ-hydroxo)bis­(μ-aqua)­tetra­nickel(II) hexa­kis­(nitrate) tetra­deca­hydrate (Marino *et al.*, 2019 ▸) and *catena*-[[μ^2^-3,6-di(pyridin-2-yl)pyridazine]bis­(μ^2^-azido)­diaza­idodicopper monohydrate] (Mastropietro *et al.*, 2013 ▸).

## Synthesis and crystallization   

6-*O*-Propargyl-1,2:3,4-di-*O*-iso­propyl­idene-α-d-galacto­pyran­oside (4 mmol) was added to a solution of 3,6-bis­(2-pyrid­yl)-1,2,4,5-tetra­zine (4 mmol) in toluene (20 ml). Stirring was continued at room temperature for 4 h. The solvent was removed under reduced pressure. The residue was separated by chromatography on a column of silica gel with ethyl acetate/hexane (1:2) as eluent. Colourless crystals were isolated on evaporation of the solvent (yield: 82%).

## Refinement   

Crystal data, data collection and structure refinement details are summarized in Table 3 ▸. Water hydrogen atoms were located in a difference-Fourier map and refined with the distance constraint O—H = 0.80 (2) Å. Other H atoms were positioned geometrically with C—H = 0.93, 0.98, 0.97 and 0.96 Å, for aromatic, methine, methyl­ene and methyl H atoms, respectively, and constrained to ride on their parent atoms, with *U*_iso_(H) = 1.5*U*_eq_(C-meth­yl) or 1.2*U*_eq_(C) for all other H atoms.

## Supplementary Material

Crystal structure: contains datablock(s) I, global. DOI: 10.1107/S2056989019009848/lh5910sup1.cif

Structure factors: contains datablock(s) I. DOI: 10.1107/S2056989019009848/lh5910Isup2.hkl

Supporting information file. DOI: 10.1107/S2056989019009848/lh5910Isup3.cdx

CCDC reference: 1939591

Additional supporting information:  crystallographic information; 3D view; checkCIF report

## Figures and Tables

**Table 1:** Hydrogen-bond geometry (Å, °) *Cg* is the centroid of the N3/C18–C22 ring.

*D*—H⋯*A*	*D*—H	H⋯*A*	*D*⋯*A*	*D*—H⋯*A*
O7—H7*A*⋯N2^i^	0.84 (2)	2.18 (3)	3.019 (4)	172 (6)
O7—H7*B*⋯O1	0.86 (2)	2.30 (3)	3.112 (4)	157 (6)
O7—H7*B*⋯O6	0.86 (2)	2.57 (5)	3.176 (5)	129 (5)
C2—H2⋯O3^ii^	0.98	2.51	3.444 (4)	160
C12—H12*A*⋯*Cg* ^iv^	0.97	3.07	3.761 (3)	130

Symmetry codes: (i) 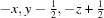; (ii) 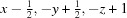; (iv) 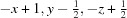.

**Table 2:** Selected interatomic distances (Å)

O1⋯O3	3.153 (2)	C2⋯C4^ii^	3.538 (4)
O1⋯O4	3.115 (3)	C2⋯H4^ii^	2.96
O1⋯O5	2.999 (3)	C3⋯H1	2.88
O1⋯O6	2.920 (3)	C4⋯H11*A*	2.84
O3⋯O1	3.153 (2)	C4⋯H2^iii^	2.83
O3⋯C1	3.002 (3)	C4⋯H1	2.76
O7⋯O1	3.112 (3)	C5⋯H9*A*	2.85
O7⋯O6	3.176 (3)	C10⋯H1	2.93
O7⋯N2^i^	3.020 (3)	H1⋯H10*C*	2.24
O2⋯H1	2.70	H2⋯H4^ii^	2.44
O2⋯H4^ii^	2.90	H4⋯H11*A*	2.47
O3⋯H1	2.54	H5⋯H9*A*	2.56
O3⋯H2^iii^	2.51	H7*A*⋯H19^i^	2.20
O5⋯H12*B*	2.70	H7*A*⋯N1^i^	2.84 (3)
O5⋯H12*A*	2.77	H7*A*⋯N2^i^	2.19 (4)
O6⋯H17	2.23	H7*B*⋯O1	2.30 (2)
O7⋯H19^i^	2.64	H7*B*⋯O6	2.56 (4)
N4⋯C13	2.776 (3)	H8*A*⋯H9*C*	2.55
N1⋯H24	2.44	H8*B*⋯H9*B*	2.50
N2⋯H19	2.56	H8*C*⋯H11*C* ^ii^	2.48
N3⋯H17	2.46	H10*A*⋯H11*C*	2.53
N4⋯H13*A*	2.56	H10*B*⋯H11*B*	2.57
N4⋯H13*B*	2.54	H12*A*⋯H13*B*	2.26
C1⋯C3	3.485 (3)		

Symmetry codes: (i) 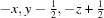; (ii) 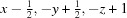; (iii) 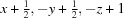.

**Table 3:** Experimental details

Crystal data	
Chemical formula	C_27_H_30_N_4_O_6_·H_2_O
*M*_r_	524.56
Crystal system, space group	Orthorhombic, *P*2_1_2_1_2_1_
Temperature (K)	150
*a*, *b*, *c* (Å)	8.8417 (3), 11.3252 (3), 25.7003 (8)
*V* (Å^3^)	2573.47 (14)
*Z*	4
Radiation type	Cu *K*α
μ (mm^−1^)	0.82
Crystal size (mm)	0.47 × 0.15 × 0.10

Data collection	
Diffractometer	Rigaku Oxford Diffraction SuperNova, single source at offset, AtlasS2
Absorption correction	Multi-scan (*CrysAlis PRO* (Rigaku OD, 2015 ▸)
*T*_min_, *T*_max_	0.656, 1.000
No. of measured, independent and observed [*I* > 2σ(*I*)] reflections	6128, 4277, 3853
*R*_int_	0.037
(sin θ/λ)_max_ (Å^−1^)	0.618

Refinement	
*R*[*F* ^2^ > 2σ(*F* ^2^)], *wR*(*F* ^2^), *S*	0.048, 0.121, 1.10
No. of reflections	4277
No. of parameters	353
No. of restraints	2
H-atom treatment	H atoms treated by a mixture of independent and constrained refinement
Δρ_max_, Δρ_min_ (e Å^−3^)	0.27, −0.36
Absolute structure	Flack *x* determined using 1226 quotients [(*I* ^+^)−(*I* ^−^)]/[(*I* ^+^)+(*I* ^−^)] (Parsons et al., 2013 ▸)
Absolute structure parameter	−0.01 (16)

Computer programs: *CrysAlis PRO* (Rigaku OD, 2015 ▸), *SHELXS97* (Sheldrick, 2008 ▸), *SHELXL2018* (Sheldrick, 2015 ▸), *ORTEP-3* for Windows and *WinGX* (Farrugia, 2012 ▸) and *PLATON* (Spek, 2015 ▸).

## supplementary crystallographic information

### Crystal data

**Table:**

C_27_H_30_N_4_O_6_·H_2_O	*D*_x_ = 1.354 Mg m^−^^3^
*M**_r_* = 524.56	Cu *K*α radiation, λ = 1.54184 Å
Orthorhombic, *P*2_1_2_1_2_1_	Cell parameters from 2843 reflections
*a* = 8.8417 (3) Å	θ = 3.3–72.3°
*b* = 11.3252 (3) Å	µ = 0.82 mm^−^^1^
*c* = 25.7003 (8) Å	*T* = 150 K
*V* = 2573.47 (14) Å^3^	Plate, colourless
*Z* = 4	0.47 × 0.15 × 0.10 mm
*F*(000) = 1112	

### Data collection

**Table:**

Rigaku Oxford Diffraction SuperNova, single source at offset, AtlasS2 diffractometer	4277 independent reflections
Radiation source: SuperNova(Cu) micro-focus sealed X-ray Source	3853 reflections with *I* > 2σ(*I*)
Detector resolution: 5.1990 pixels mm^-1^	*R*_int_ = 0.037
ω scans	θ_max_ = 72.4°, θ_min_ = 3.4°
Absorption correction: multi-scan (CrysAlis PRO (Rigaku OD, 2015)	*h* = −9→10
*T*_min_ = 0.656, *T*_max_ = 1.000	*k* = −13→5
6128 measured reflections	*l* = −31→29

### Refinement

**Table:**

Refinement on *F*^2^	Hydrogen site location: mixed
Least-squares matrix: full	H atoms treated by a mixture of independent and constrained refinement
*R*[*F*^2^ > 2σ(*F*^2^)] = 0.048	*w* = 1/[σ^2^(*F*_o_^2^) + (0.0538*P*)^2^ + 0.4885*P*] where *P* = (*F*_o_^2^ + 2*F*_c_^2^)/3
*wR*(*F*^2^) = 0.121	(Δ/σ)_max_ < 0.001
*S* = 1.10	Δρ_max_ = 0.27 e Å^−^^3^
4277 reflections	Δρ_min_ = −0.36 e Å^−^^3^
353 parameters	Absolute structure: Flack *x* determined using 1226 quotients [(*I*^+^)-(*I*^-^)]/[(*I*^+^)+(*I*^-^)] (Parsons et al., 2013)
2 restraints	Absolute structure parameter: −0.01 (16)

### Special details

**Table:**

Geometry. All esds (except the esd in the dihedral angle between two l.s. planes) are estimated using the full covariance matrix. The cell esds are taken into account individually in the estimation of esds in distances, angles and torsion angles; correlations between esds in cell parameters are only used when they are defined by crystal symmetry. An approximate (isotropic) treatment of cell esds is used for estimating esds involving l.s. planes.

### Fractional atomic coordinates and isotropic or equivalent isotropic displacement parameters (Å^2^)

**Table:**

	*x*	*y*	*z*	*U*_iso_*/*U*_eq_	
O1	0.3541 (3)	0.1622 (2)	0.41689 (9)	0.0282 (5)	
O2	0.3948 (3)	0.34989 (19)	0.45130 (10)	0.0314 (5)	
O3	0.6462 (3)	0.30998 (18)	0.44345 (9)	0.0275 (5)	
O4	0.5889 (3)	0.00085 (19)	0.47272 (8)	0.0298 (5)	
O5	0.5504 (4)	−0.04337 (19)	0.38768 (10)	0.0397 (7)	
O6	0.2874 (3)	0.20336 (19)	0.30710 (9)	0.0341 (6)	
N1	0.0414 (3)	0.4001 (2)	0.16743 (11)	0.0279 (6)	
N2	0.0957 (3)	0.5016 (2)	0.18697 (10)	0.0280 (6)	
N3	0.3589 (4)	0.6013 (3)	0.28330 (11)	0.0324 (6)	
N4	0.0746 (4)	0.0864 (2)	0.17094 (12)	0.0358 (7)	
C1	0.4587 (4)	0.1547 (3)	0.37413 (12)	0.0258 (7)	
H1	0.495093	0.234348	0.366098	0.031*	
C2	0.4106 (4)	0.2260 (3)	0.45945 (13)	0.0277 (7)	
H2	0.355033	0.203400	0.490891	0.033*	
C3	0.5408 (4)	0.4049 (3)	0.44788 (14)	0.0310 (7)	
C4	0.5790 (4)	0.2100 (3)	0.46886 (12)	0.0246 (6)	
H4	0.600843	0.212023	0.506234	0.030*	
C5	0.6463 (4)	0.0999 (3)	0.44448 (12)	0.0254 (6)	
H5	0.756973	0.102135	0.446060	0.031*	
C6	0.5926 (4)	0.0790 (3)	0.38885 (12)	0.0272 (7)	
H6	0.676097	0.092996	0.364527	0.033*	
C7	0.5754 (4)	−0.0947 (3)	0.43729 (13)	0.0310 (7)	
C8	0.4413 (5)	−0.1669 (4)	0.4525 (2)	0.0550 (12)	
H8A	0.453499	−0.194150	0.487614	0.082*	
H8B	0.432577	−0.233440	0.429569	0.082*	
H8C	0.351559	−0.119463	0.450095	0.082*	
C9	0.7193 (5)	−0.1667 (3)	0.43584 (15)	0.0394 (9)	
H9A	0.802843	−0.116353	0.427016	0.059*	
H9B	0.709878	−0.227881	0.410208	0.059*	
H9C	0.736583	−0.201467	0.469376	0.059*	
C10	0.5503 (5)	0.4786 (3)	0.39910 (15)	0.0427 (9)	
H10A	0.650263	0.510885	0.395892	0.064*	
H10B	0.478045	0.541722	0.400967	0.064*	
H10C	0.528653	0.430067	0.369395	0.064*	
C11	0.5698 (5)	0.4759 (3)	0.49680 (16)	0.0442 (9)	
H11A	0.569729	0.424125	0.526380	0.066*	
H11B	0.491803	0.534147	0.500856	0.066*	
H11C	0.666221	0.514400	0.494189	0.066*	
C12	0.3729 (4)	0.1086 (3)	0.32779 (13)	0.0286 (7)	
H12A	0.442659	0.078782	0.301797	0.034*	
H12B	0.306342	0.044768	0.338237	0.034*	
C13	0.2122 (4)	0.1736 (3)	0.26014 (13)	0.0275 (7)	
H13A	0.122273	0.127514	0.267550	0.033*	
H13B	0.278292	0.127130	0.238042	0.033*	
C14	0.1697 (4)	0.2873 (3)	0.23325 (12)	0.0253 (6)	
C15	0.0791 (4)	0.2958 (3)	0.18820 (12)	0.0247 (6)	
C16	0.1872 (4)	0.4980 (3)	0.22846 (12)	0.0247 (6)	
C17	0.2238 (4)	0.3919 (3)	0.25319 (12)	0.0263 (6)	
H17	0.284224	0.391984	0.282822	0.032*	
C18	0.2537 (4)	0.6114 (3)	0.24658 (12)	0.0265 (7)	
C19	0.2107 (4)	0.7197 (3)	0.22546 (14)	0.0315 (7)	
H19	0.135761	0.723846	0.200145	0.038*	
C20	0.2823 (5)	0.8210 (3)	0.24304 (15)	0.0380 (8)	
H20	0.256630	0.894499	0.229490	0.046*	
C21	0.3920 (4)	0.8114 (3)	0.28088 (15)	0.0376 (9)	
H21	0.441630	0.877961	0.293426	0.045*	
C22	0.4265 (5)	0.7001 (3)	0.29975 (14)	0.0349 (8)	
H22	0.500690	0.693707	0.325247	0.042*	
C23	0.0175 (4)	0.1927 (3)	0.15887 (12)	0.0253 (7)	
C24	−0.0897 (4)	0.2085 (3)	0.12003 (13)	0.0325 (7)	
H24	−0.126828	0.283313	0.112401	0.039*	
C25	−0.1402 (5)	0.1107 (3)	0.09294 (14)	0.0378 (8)	
H25	−0.211674	0.119110	0.066653	0.045*	
C26	−0.0836 (5)	0.0004 (3)	0.10520 (14)	0.0375 (8)	
H26	−0.116014	−0.066836	0.087656	0.045*	
C27	0.0224 (5)	−0.0065 (3)	0.14423 (15)	0.0414 (9)	
H27	0.060342	−0.080694	0.152640	0.050*	
O7	0.0146 (4)	0.1893 (3)	0.38725 (14)	0.0542 (8)	
H7A	−0.011 (7)	0.141 (5)	0.3642 (19)	0.081*	
H7B	0.111 (3)	0.189 (6)	0.386 (2)	0.081*	

### Atomic displacement parameters (Å^2^)

**Table:**

	*U*^11^	*U*^22^	*U*^33^	*U*^12^	*U*^13^	*U*^23^
O1	0.0250 (11)	0.0272 (10)	0.0325 (11)	−0.0023 (10)	−0.0016 (10)	−0.0023 (9)
O2	0.0294 (12)	0.0210 (10)	0.0438 (13)	0.0057 (9)	−0.0026 (11)	−0.0024 (9)
O3	0.0280 (11)	0.0181 (10)	0.0364 (12)	0.0007 (9)	0.0010 (10)	0.0006 (9)
O4	0.0419 (14)	0.0190 (9)	0.0286 (11)	−0.0011 (10)	−0.0005 (11)	0.0020 (8)
O5	0.0627 (18)	0.0179 (10)	0.0385 (13)	0.0041 (11)	−0.0184 (14)	−0.0038 (9)
O6	0.0499 (15)	0.0190 (9)	0.0333 (12)	0.0033 (11)	−0.0182 (12)	−0.0023 (9)
N1	0.0324 (15)	0.0203 (12)	0.0310 (13)	−0.0004 (11)	−0.0037 (13)	−0.0002 (10)
N2	0.0339 (16)	0.0192 (11)	0.0310 (13)	0.0005 (11)	0.0006 (13)	−0.0010 (10)
N3	0.0367 (16)	0.0252 (13)	0.0352 (14)	−0.0026 (12)	−0.0011 (13)	−0.0045 (11)
N4	0.0485 (18)	0.0212 (12)	0.0377 (15)	0.0021 (13)	−0.0154 (15)	−0.0018 (11)
C1	0.0331 (17)	0.0180 (12)	0.0265 (15)	−0.0010 (13)	−0.0025 (14)	0.0020 (11)
C2	0.0333 (17)	0.0201 (13)	0.0298 (15)	0.0016 (13)	0.0036 (15)	−0.0006 (12)
C3	0.0324 (18)	0.0192 (13)	0.0415 (18)	0.0039 (14)	−0.0005 (16)	−0.0011 (13)
C4	0.0299 (16)	0.0179 (12)	0.0260 (14)	−0.0003 (13)	−0.0018 (14)	−0.0005 (11)
C5	0.0295 (16)	0.0188 (13)	0.0280 (15)	0.0026 (13)	0.0008 (14)	0.0019 (12)
C6	0.0333 (18)	0.0209 (13)	0.0274 (15)	0.0010 (13)	−0.0001 (14)	−0.0005 (12)
C7	0.0382 (19)	0.0194 (13)	0.0354 (17)	0.0006 (14)	−0.0015 (16)	−0.0004 (12)
C8	0.047 (2)	0.0365 (19)	0.081 (3)	−0.012 (2)	0.017 (2)	−0.020 (2)
C9	0.044 (2)	0.0321 (17)	0.042 (2)	0.0111 (17)	−0.0034 (18)	0.0023 (15)
C10	0.051 (2)	0.0257 (16)	0.051 (2)	0.0042 (16)	0.002 (2)	0.0079 (15)
C11	0.051 (2)	0.0317 (17)	0.050 (2)	0.0028 (18)	−0.007 (2)	−0.0124 (16)
C12	0.0360 (18)	0.0191 (12)	0.0307 (15)	0.0030 (13)	−0.0085 (15)	0.0024 (12)
C13	0.0331 (17)	0.0174 (13)	0.0319 (16)	−0.0019 (13)	−0.0062 (14)	0.0005 (12)
C14	0.0270 (16)	0.0224 (14)	0.0265 (15)	0.0009 (13)	−0.0004 (13)	0.0014 (12)
C15	0.0269 (16)	0.0194 (13)	0.0277 (14)	0.0000 (13)	−0.0005 (14)	−0.0013 (12)
C16	0.0265 (16)	0.0212 (13)	0.0265 (14)	−0.0003 (12)	0.0026 (13)	−0.0020 (11)
C17	0.0297 (16)	0.0220 (13)	0.0272 (15)	0.0006 (13)	−0.0020 (14)	−0.0011 (12)
C18	0.0285 (16)	0.0217 (14)	0.0293 (15)	−0.0001 (12)	0.0037 (14)	−0.0029 (12)
C19	0.0348 (18)	0.0208 (14)	0.0389 (17)	−0.0005 (15)	−0.0010 (16)	−0.0021 (13)
C20	0.043 (2)	0.0209 (15)	0.050 (2)	−0.0014 (15)	0.0039 (18)	−0.0021 (14)
C21	0.039 (2)	0.0247 (15)	0.049 (2)	−0.0039 (14)	0.0037 (18)	−0.0108 (14)
C22	0.0395 (19)	0.0276 (15)	0.0375 (17)	−0.0033 (16)	−0.0026 (16)	−0.0073 (14)
C23	0.0262 (16)	0.0238 (14)	0.0259 (14)	−0.0037 (12)	0.0008 (13)	0.0003 (12)
C24	0.0327 (18)	0.0295 (15)	0.0353 (16)	0.0029 (15)	−0.0075 (15)	−0.0002 (14)
C25	0.0383 (19)	0.0395 (18)	0.0358 (18)	−0.0010 (17)	−0.0148 (17)	−0.0026 (15)
C26	0.049 (2)	0.0271 (15)	0.0366 (17)	−0.0079 (17)	−0.0059 (18)	−0.0080 (14)
C27	0.058 (3)	0.0234 (15)	0.0428 (19)	0.0013 (16)	−0.015 (2)	−0.0027 (15)
O7	0.0524 (18)	0.0479 (16)	0.0623 (19)	0.0030 (15)	−0.0015 (16)	−0.0140 (14)

### Geometric parameters (Å, º)

**Table:**

O1—C2	1.403 (4)	C9—H9B	0.9600
O1—C1	1.439 (4)	C9—H9C	0.9600
O2—C2	1.425 (4)	C10—H10A	0.9600
O2—C3	1.437 (4)	C10—H10B	0.9600
O3—C3	1.428 (4)	C10—H10C	0.9600
O3—C4	1.436 (4)	C11—H11A	0.9600
O4—C7	1.419 (4)	C11—H11B	0.9600
O4—C5	1.430 (4)	C11—H11C	0.9600
O5—C7	1.418 (4)	C12—H12A	0.9700
O5—C6	1.436 (4)	C12—H12B	0.9700
O6—C12	1.416 (4)	C13—C14	1.509 (4)
O6—C13	1.418 (4)	C13—H13A	0.9700
N1—C15	1.339 (4)	C13—H13B	0.9700
N1—N2	1.343 (4)	C14—C17	1.377 (4)
N2—C16	1.339 (4)	C14—C15	1.411 (4)
N3—C18	1.330 (5)	C15—C23	1.493 (4)
N3—C22	1.337 (5)	C16—C17	1.397 (4)
N4—C27	1.339 (5)	C16—C18	1.488 (4)
N4—C23	1.341 (4)	C17—H17	0.9300
C1—C12	1.506 (5)	C18—C19	1.394 (5)
C1—C6	1.510 (5)	C19—C20	1.386 (5)
C1—H1	0.9800	C19—H19	0.9300
C2—C4	1.519 (5)	C20—C21	1.378 (6)
C2—H2	0.9800	C20—H20	0.9300
C3—C10	1.508 (5)	C21—C22	1.385 (5)
C3—C11	1.514 (5)	C21—H21	0.9300
C4—C5	1.517 (4)	C22—H22	0.9300
C4—H4	0.9800	C23—C24	1.388 (5)
C5—C6	1.525 (4)	C24—C25	1.382 (5)
C5—H5	0.9800	C24—H24	0.9300
C6—H6	0.9800	C25—C26	1.383 (5)
C7—C8	1.492 (6)	C25—H25	0.9300
C7—C9	1.512 (5)	C26—C27	1.375 (6)
C8—H8A	0.9600	C26—H26	0.9300
C8—H8B	0.9600	C27—H27	0.9300
C8—H8C	0.9600	O7—H7A	0.84 (2)
C9—H9A	0.9600	O7—H7B	0.86 (2)
			
O1···O3	3.153 (2)	C2···C4^ii^	3.538 (4)
O1···O4	3.115 (3)	C2···H4^ii^	2.96
O1···O5	2.999 (3)	C3···H1	2.88
O1···O6	2.920 (3)	C4···H11A	2.84
O3···O1	3.153 (2)	C4···H2^iii^	2.83
O3···C1	3.002 (3)	C4···H1	2.76
O7···O1	3.112 (3)	C5···H9A	2.85
O7···O6	3.176 (3)	C10···H1	2.93
O7···N2^i^	3.020 (3)	H1···H10C	2.24
O2···H1	2.70	H2···H4^ii^	2.44
O2···H4^ii^	2.90	H4···H11A	2.47
O3···H1	2.54	H5···H9A	2.56
O3···H2^iii^	2.51	H7A···H19^i^	2.20
O5···H12B	2.70	H7A···N1^i^	2.84 (3)
O5···H12A	2.77	H7A···N2^i^	2.19 (4)
O6···H17	2.23	H7B···O1	2.30 (2)
O7···H19^i^	2.64	H7B···O6	2.56 (4)
N4···C13	2.776 (3)	H8A···H9C	2.55
N1···H24	2.44	H8B···H9B	2.50
N2···H19	2.56	H8C···H11C^ii^	2.48
N3···H17	2.46	H10A···H11C	2.53
N4···H13A	2.56	H10B···H11B	2.57
N4···H13B	2.54	H12A···H13B	2.26
C1···C3	3.485 (3)		
			
C2—O1—C1	113.4 (2)	H10A—C10—H10B	109.5
C2—O2—C3	110.4 (2)	C3—C10—H10C	109.5
C3—O3—C4	106.7 (2)	H10A—C10—H10C	109.5
C7—O4—C5	107.6 (2)	H10B—C10—H10C	109.5
C7—O5—C6	109.6 (2)	C3—C11—H11A	109.5
C12—O6—C13	112.9 (2)	C3—C11—H11B	109.5
C15—N1—N2	121.1 (3)	H11A—C11—H11B	109.5
C16—N2—N1	119.2 (3)	C3—C11—H11C	109.5
C18—N3—C22	117.7 (3)	H11A—C11—H11C	109.5
C27—N4—C23	117.2 (3)	H11B—C11—H11C	109.5
O1—C1—C12	107.5 (3)	O6—C12—C1	107.7 (2)
O1—C1—C6	110.3 (2)	O6—C12—H12A	110.2
C12—C1—C6	113.4 (3)	C1—C12—H12A	110.2
O1—C1—H1	108.5	O6—C12—H12B	110.2
C12—C1—H1	108.5	C1—C12—H12B	110.2
C6—C1—H1	108.5	H12A—C12—H12B	108.5
O1—C2—O2	111.0 (3)	O6—C13—C14	107.7 (2)
O1—C2—C4	114.3 (3)	O6—C13—H13A	110.2
O2—C2—C4	103.7 (3)	C14—C13—H13A	110.2
O1—C2—H2	109.2	O6—C13—H13B	110.2
O2—C2—H2	109.2	C14—C13—H13B	110.2
C4—C2—H2	109.2	H13A—C13—H13B	108.5
O3—C3—O2	105.4 (2)	C17—C14—C15	116.4 (3)
O3—C3—C10	108.3 (3)	C17—C14—C13	118.5 (3)
O2—C3—C10	109.9 (3)	C15—C14—C13	125.1 (3)
O3—C3—C11	110.8 (3)	N1—C15—C14	121.9 (3)
O2—C3—C11	109.4 (3)	N1—C15—C23	113.5 (3)
C10—C3—C11	112.8 (3)	C14—C15—C23	124.6 (3)
O3—C4—C5	107.3 (2)	N2—C16—C17	121.9 (3)
O3—C4—C2	103.9 (2)	N2—C16—C18	117.5 (3)
C5—C4—C2	114.6 (3)	C17—C16—C18	120.5 (3)
O3—C4—H4	110.3	C14—C17—C16	119.3 (3)
C5—C4—H4	110.3	C14—C17—H17	120.3
C2—C4—H4	110.3	C16—C17—H17	120.3
O4—C5—C4	107.2 (2)	N3—C18—C19	122.9 (3)
O4—C5—C6	104.1 (2)	N3—C18—C16	115.1 (3)
C4—C5—C6	113.2 (3)	C19—C18—C16	122.0 (3)
O4—C5—H5	110.7	C20—C19—C18	118.5 (3)
C4—C5—H5	110.7	C20—C19—H19	120.8
C6—C5—H5	110.7	C18—C19—H19	120.8
O5—C6—C1	109.8 (3)	C21—C20—C19	119.1 (3)
O5—C6—C5	104.5 (2)	C21—C20—H20	120.5
C1—C6—C5	113.0 (3)	C19—C20—H20	120.5
O5—C6—H6	109.8	C20—C21—C22	118.3 (3)
C1—C6—H6	109.8	C20—C21—H21	120.8
C5—C6—H6	109.8	C22—C21—H21	120.8
O5—C7—O4	106.1 (2)	N3—C22—C21	123.5 (3)
O5—C7—C8	109.6 (4)	N3—C22—H22	118.2
O4—C7—C8	108.4 (3)	C21—C22—H22	118.2
O5—C7—C9	109.3 (3)	N4—C23—C24	122.6 (3)
O4—C7—C9	110.9 (3)	N4—C23—C15	116.6 (3)
C8—C7—C9	112.3 (3)	C24—C23—C15	120.8 (3)
C7—C8—H8A	109.5	C25—C24—C23	118.7 (3)
C7—C8—H8B	109.5	C25—C24—H24	120.7
H8A—C8—H8B	109.5	C23—C24—H24	120.7
C7—C8—H8C	109.5	C24—C25—C26	119.5 (3)
H8A—C8—H8C	109.5	C24—C25—H25	120.3
H8B—C8—H8C	109.5	C26—C25—H25	120.3
C7—C9—H9A	109.5	C27—C26—C25	117.7 (3)
C7—C9—H9B	109.5	C27—C26—H26	121.2
H9A—C9—H9B	109.5	C25—C26—H26	121.2
C7—C9—H9C	109.5	N4—C27—C26	124.4 (3)
H9A—C9—H9C	109.5	N4—C27—H27	117.8
H9B—C9—H9C	109.5	C26—C27—H27	117.8
C3—C10—H10A	109.5	H7A—O7—H7B	104 (6)
C3—C10—H10B	109.5		
			
C15—N1—N2—C16	−1.1 (5)	O1—C1—C12—O6	77.8 (3)
C2—O1—C1—C12	−167.7 (2)	C6—C1—C12—O6	−160.1 (3)
C2—O1—C1—C6	68.2 (3)	C12—O6—C13—C14	−161.7 (3)
C1—O1—C2—O2	81.1 (3)	O6—C13—C14—C17	8.0 (4)
C1—O1—C2—C4	−35.8 (3)	O6—C13—C14—C15	−172.8 (3)
C3—O2—C2—O1	−115.1 (3)	N2—N1—C15—C14	3.9 (5)
C3—O2—C2—C4	8.1 (3)	N2—N1—C15—C23	−175.6 (3)
C4—O3—C3—O2	−27.7 (3)	C17—C14—C15—N1	−3.3 (5)
C4—O3—C3—C10	−145.2 (3)	C13—C14—C15—N1	177.6 (3)
C4—O3—C3—C11	90.5 (3)	C17—C14—C15—C23	176.1 (3)
C2—O2—C3—O3	11.5 (4)	C13—C14—C15—C23	−3.0 (5)
C2—O2—C3—C10	128.0 (3)	N1—N2—C16—C17	−2.2 (5)
C2—O2—C3—C11	−107.7 (3)	N1—N2—C16—C18	175.7 (3)
C3—O3—C4—C5	154.1 (3)	C15—C14—C17—C16	0.0 (5)
C3—O3—C4—C2	32.4 (3)	C13—C14—C17—C16	179.2 (3)
O1—C2—C4—O3	96.5 (3)	N2—C16—C17—C14	2.7 (5)
O2—C2—C4—O3	−24.4 (3)	C18—C16—C17—C14	−175.2 (3)
O1—C2—C4—C5	−20.2 (4)	C22—N3—C18—C19	−0.8 (5)
O2—C2—C4—C5	−141.2 (3)	C22—N3—C18—C16	177.7 (3)
C7—O4—C5—C4	147.7 (3)	N2—C16—C18—N3	−171.4 (3)
C7—O4—C5—C6	27.5 (3)	C17—C16—C18—N3	6.5 (4)
O3—C4—C5—O4	175.2 (2)	N2—C16—C18—C19	7.1 (5)
C2—C4—C5—O4	−70.0 (3)	C17—C16—C18—C19	−174.9 (3)
O3—C4—C5—C6	−70.5 (3)	N3—C18—C19—C20	0.9 (5)
C2—C4—C5—C6	44.2 (4)	C16—C18—C19—C20	−177.5 (3)
C7—O5—C6—C1	−121.7 (3)	C18—C19—C20—C21	−0.5 (5)
C7—O5—C6—C5	−0.3 (4)	C19—C20—C21—C22	0.1 (5)
O1—C1—C6—O5	76.1 (3)	C18—N3—C22—C21	0.4 (5)
C12—C1—C6—O5	−44.5 (4)	C20—C21—C22—N3	−0.1 (6)
O1—C1—C6—C5	−40.1 (3)	C27—N4—C23—C24	−0.9 (5)
C12—C1—C6—C5	−160.7 (3)	C27—N4—C23—C15	−178.8 (3)
O4—C5—C6—O5	−16.4 (3)	N1—C15—C23—N4	167.1 (3)
C4—C5—C6—O5	−132.5 (3)	C14—C15—C23—N4	−12.4 (5)
O4—C5—C6—C1	102.9 (3)	N1—C15—C23—C24	−10.8 (4)
C4—C5—C6—C1	−13.2 (4)	C14—C15—C23—C24	169.7 (3)
C6—O5—C7—O4	17.2 (4)	N4—C23—C24—C25	0.4 (5)
C6—O5—C7—C8	134.1 (3)	C15—C23—C24—C25	178.2 (3)
C6—O5—C7—C9	−102.4 (3)	C23—C24—C25—C26	0.2 (6)
C5—O4—C7—O5	−28.3 (4)	C24—C25—C26—C27	−0.3 (6)
C5—O4—C7—C8	−146.0 (3)	C23—N4—C27—C26	0.8 (6)
C5—O4—C7—C9	90.3 (3)	C25—C26—C27—N4	−0.3 (7)
C13—O6—C12—C1	174.4 (3)		

Symmetry codes: (i) −*x*, *y*−1/2, −*z*+1/2; (ii) *x*−1/2, −*y*+1/2, −*z*+1; (iii) *x*+1/2, −*y*+1/2, −*z*+1.

### Hydrogen-bond geometry (Å, º)

Cg is the centroid of the N3/C18–C22 ring.

**Table:**

*D*—H···*A*	*D*—H	H···*A*	*D*···*A*	*D*—H···*A*
O7—H7*A*···N2^i^	0.84 (2)	2.18 (3)	3.019 (4)	172 (6)
O7—H7*B*···O1	0.86 (2)	2.30 (3)	3.112 (4)	157 (6)
O7—H7*B*···O6	0.86 (2)	2.57 (5)	3.176 (5)	129 (5)
C2—H2···O3^ii^	0.98	2.51	3.444 (4)	160
C12—H12*A*···*Cg*^iv^	0.97	3.07	3.761 (3)	130

Symmetry codes: (i) −*x*, *y*−1/2, −*z*+1/2; (ii) *x*−1/2, −*y*+1/2, −*z*+1; (iv) −*x*+1, *y*−1/2, −*z*+1/2.
